# Extensive pulmonary embolism as complication of oligosymptomatic COVID-19: case report

**DOI:** 10.1590/1677-5449.200239

**Published:** 2021-09-24

**Authors:** Ailton Carvalho Barbosa, Lívia Silva de Paula Faria, Larissa Freitas Peixoto Gloria, Graziella Viana da Silva, Paulo Henrique Ribeiro de Oliveira, Fabiano Vieira de Araujo, Felipe Eulalio Baldi Pessanha

**Affiliations:** 1 Hospital Estadual Ferreira Machado, Campos dos Goytacazes, RJ, Brasil; 2 Centro Universitário de Volta Redonda, Volta Redonda, RJ, Brasil

**Keywords:** covid‐19, pulmonary embolism, deep vein thrombosis

## Abstract

Although the pathophysiology of coagulopathy associated with the 2019 coronavirus disease (COVID-19) is not well known, occurrence of pulmonary embolism (PE) is frequently observed. However, few cases have been described in the literature in which patients who had asymptomatic COVID-19, with no risk factors for venous thromboembolism (VTE), presented extensive acute PE. We report the case of a patient with asymptomatic COVID-19, complicated by deep vein thrombosis and later by extensive acute PE, suggesting that these conditions should be systematically considered, even in asymptomatic COVID-19 patients with no known risk factors for VTE.

## INTRODUCTION

The coronavirus 2019 disease (COVID-19) pandemic, caused by the severe acute respiratory syndrome coronavirus 2 (SARS-CoV-2), has provoked major difficulties for health systems because of the complete lack of knowledge about the disease, from its etiopathogenesis and complications to its treatment.^1^ Although the pathophysiology of coagulopathy associated with COVID‐19 is not yet fully understood, it is known that there is a high risk of the disease complicating with pulmonary embolism (PE), primarily in severe and critical cases. The mechanism of pathogenesis appears to be related to a hypercoagulable state caused by the massive release of inflammatory cytokines in the body.^2^ However, the incidence of cases of extensive acute PE as a complication of oligosymptomatic COVID-19 in patients with no known risk factors for venous thromboembolism (VTE) has not yet been determined.

In this case report, we describe a patient who was suspected of having oligosymptomatic COVID-19 and had no known risk factors for VTE, who developed deep venous thrombosis (DVT) and then progressed to extensive acute PE. This case suggests that these conditions should possibly be considered systematically, even in oligosymptomatic patients without known risk factors for VTE. This study was duly approved by the Research Ethics Committee (CAAE 43681721.3.0000.5237, protocol 4.641.084).

## CASE DESCRIPTION

The patient was a 57-year-old male with no history of systemic arterial hypertension, diabetes mellitus, or other comorbidities, who sought care at the emergency room complaining of mild dyspnea and persistent edema of the right lower limb. He reported that the edema had had onset approximately 1 month previously, he had applied local compression and ice, and had concurrently had fever with shivering for 3 consecutive days. He was free from fever when examined and said he had not had coughing, headaches, or anosmia. He stated that he did not smoke or drink and had had no surgery recently.

On physical examination, the patient was in good general health, free from fever, and dyspneic in room air. His body mass index was 27.78 kg/m^2^. Blood pressure was 120×70 mmHg, heart rate was 90 bpm, and respiratory rate was 20 irpm, with 98% oxygen saturation in room air. Respiratory and cardiac auscultation findings were normal. The right lower limb had edema (++/4+), without muscle clubbing, and pulses were palpable bilaterally. Laboratory test results included a 12,150 white blood count, without left shift, 41% hematocrit, normal CK-MB, and electrolytes, urea, and creatinine were all within normal limits.

**Figure 1:**
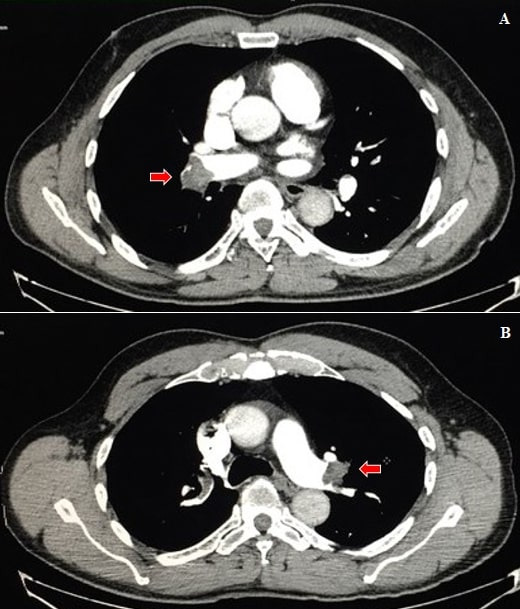
Computed tomography angiography of the chest, axial plane, showing failure to completely fill right **(A)** and left **(B)** pulmonary arteries because of obstruction by thrombus (arrows).

**Figure 2:**
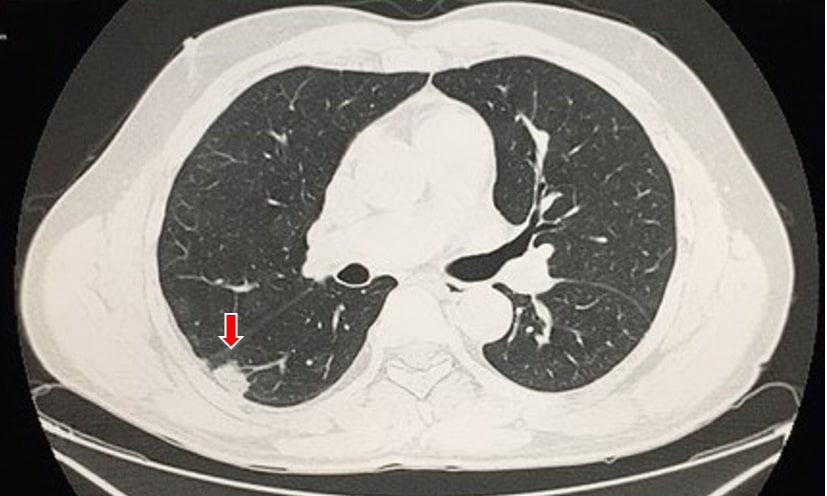
Computed tomography angiography of the chest, with pulmonary parenchyma window, showing area of pulmonary infarction on the right (arrow).

Echocardiogram, electrocardiogram, and chest X-ray were all unremarkable. Color Doppler ultrasonography of the lower limbs was conducted to investigate the etiology of the edema, showing the right popliteal and soleus veins with signs of acute venous thrombosis (incompressible veins, with intraluminal echogenic material and no flow on Doppler). D-dimer (DD) and C-reactive protein (CRP) tests were not available at that time. In order to try to explain the dyspnea, computed tomography angiography of the chest (CTA) was conducted, which found no signs of pneumonia, but did show thrombus in the pulmonary artery bilaterally (Figure 1) with areas of pulmonary infarction on the right (Figure 2). A rapid COVID-19 test was positive for immunoglobulin G (IgG) and negative for immunoglobulin M (IgM). Based on these findings, the diagnostic hypothesis was extensive acute PE as a complication of oligosymptomatic COVID-19.

**Figure 3:**
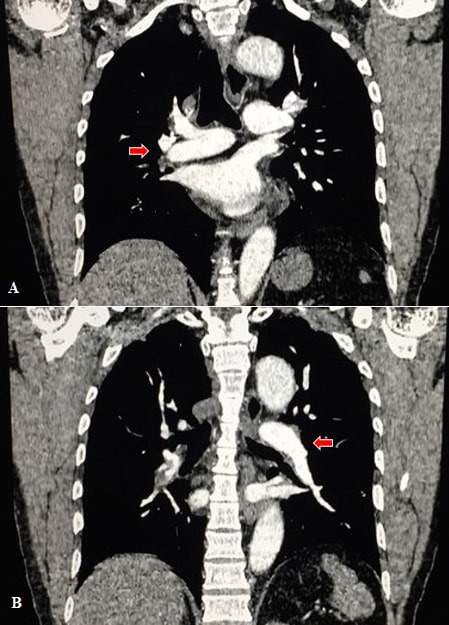
Computed tomography angiography of the chest, coronal plane, showing restored blood flow to right **(A)** and left **(B)** pulmonary arteries and removal of a large proportion of the thrombus.

After conducting an evaluation of the risks and benefits, in terms of the patient’s age and preexisting diseases, thrombolytic treatment was initiated with intravenous Alteplase, with an initial dose of 10 mg diluted in 10 mL of 0.9% saline via continuous infusion for 5 minutes, followed by 90 mg diluted in 90 mL of 0.9% saline for 2 hours intravenously. The patient remained stable throughout, with no hemodynamic instability and no complications related to use of the fibrinolytic agent. After completing administration of the Alteplase, it was observed that the dyspnea had improved. A repeat chest CTA was performed (Figure 3), 9 hours after the first, showing good restoration of flow and resolution of a large proportion of the thrombus.

While still in hospital, the patient was kept on rigorous hemodynamic monitoring and 48 hours after admission he was put on dual antiaggregation with acetylsalicylic acid 100 mg/day and clopidogrel 75 mg/day, which was had not been initiated earlier because of the risk of bleeding after use of the fibrinolytic. The patient was discharged after 6 days in hospital since it was decided not to keep him in hospital longer because of the risk of SARS-CoV-2 re-infection and because beds were needed for patients in critical conditions. Clinics run by the Brazilian National Health Service (SUS - Sistema Único de Saúde) were suspended indefinitely and so it was not possible to monitor the possible in outpatients follow-up. However, he was instructed to maintain dual antiaggregation and return to the emergency room if he noticed any warning signs or clinical deterioration.

## DISCUSSION

The case described highlights the risk of thromboembolic complications in patients diagnosed with COVID-19, even its oligosymptomatic form, and the need to remain alert to this possibility. In the case described, investigation of PE was obligatory since the patient exhibited classic signs and symptoms of DVT combined with dyspnea. The Wells score can be used to estimate risk in patients with suspected DVT and/or PE and to help guide the choice of tests and examinations in each case.^3^ According to a meta-analysis by Suh et al.,^4^ the incidence rates of PE and DVT in patients with COVID-19 were 16.5% and 14.8%, respectively. However, DVT was present in just 42.4% of the patients with PE, which is lower than the normal prevalence in patients not infected by SARS-CoV-2, which is 60%.^4^

At public services, such as the hospital at which this case was seen, the unavailability of certain tests, such as DD and CRP, can limit the ability to determine the severity and prognosis of the systemic inflammation caused by COVID-19. However, in the case described, CTA of the chest enabled the PE to be diagnosed and treatment initiated before the patient progressed to hemodynamic instability or death. Moreover, the rapid test for COVID-19 indicated that infection by SARS-CoV-2 was a possible etiology of this complication.

With regard to treatment, since SARS-CoV-2 infection is a new disease, for which there is not yet any treatment with proven efficacy, in the case described, the decision was taken to only treat the PE to prevent progression or relapse. In order to facilitate the decision on the best treatment to choose, patients should be assessed for the severity of the event. The score mentioned in the 2019 European Society of Cardiology guidelines is the PE severity index (PESI).^5^ The PESI score for the patient described was 67 points, which puts him in the class II, or low risk, category with 30-day mortality of 1.7 to 3.5%.

The Covid-19 pandemic has generated new interest in fibrinolytic agents and their possible benefits for treatment of patients with PE as a complication of SARS-CoV-2 infection.^2^ Use of these agents is justified by the intense inflammatory reaction and the major increase in cytokines that occur in COVID-19, leading to deposition of fibrin in the air spaces and lungs, which is a factor indicative of severity in the course of the disease.^2^ Nevertheless, according to the European Society of Cardiology,^5^ use of these agents in low-risk patients is only recommended as a salvage therapy if hemodynamic deterioration occurs despite prior treatment with anticoagulants.

In the case described, the medical team decided to initiate treatment with Alteplase despite the patient being hemodynamically stable for the following reasons. First, because COVID-19 is a new disease that can cause unpredictable morbid outcomes, which is why the team targeted rapid resolution of the PE. The second reason was that CTA showed that the patient had extensive PE bilaterally, for which anticoagulation would not have been as effective as a fibrinolytic to resolve the thrombi. Finally, the patient had low socioeconomic status and a low educational level, so there was no guarantee that he would continue treatment with an anticoagulant at home. For these reasons, after careful evaluation of the risks and benefits, the decision was taken to use the antifibrinolytic agent, with rigorous monitoring of the patient in a hospital setting.

After a case of PE, patients are at high risk of recurrence if continuous anticoagulation is not maintained. The American College of Chest Physicians^6^ recommends using anticoagulants for at least 3 months after a case of PE has been treated, with the duration of use varying according to the risk of bleeding and/or whether other factors that potentially contribute to the etiology of PE are still present. However, for this to be done, it is necessary to maintain regular follow-up after hospital discharge to control the international normalized ratio and guarantee that the patient is adhering to the treatment and has not developed contraindications to the treatment. As already mentioned, since outpatients follow-up would not be possible, it was decided to only use duel platelet antiaggregation with acetylsalicylic acid and clopidogrel because of the risks that anticoagulant without regular medical assessments could cause the patient.

Few cases have been described in the literature of patients who had oligosymptomatic COVID-19 and no known risk factor for thromboembolic disease and then exhibited extensive acute PE.^7^^-^10 We emphasize that while the Covid-19 pandemic is ongoing, in cases diagnosed with SARS-CoV-2 infection, whether symptomatic or asymptomatic, the possibility that the disease can complicate with a coagulopathy should be considered in cases admitted to the emergency department with DVT or PE. Therefore, primarily in patients without risk factors for disease thromboembolic, a diagnostic hypothesis that this could be a complication of COVID-19 should be considered.
