# Supplementary material for: Extensive pulmonary embolism as complication of oligosymptomatic COVID-19: case report
Source: J Vasc Bras. 2021 Sep 24;20:e20200239. doi: 10.1590/1677-5449.200239 (PMC9045534; doi:10.1590/1677-5449.200239)
Supplement: Translation

# Translation: Embolia pulmonar aguda extensa como complicação de COVID-19 oligossintomática: relato de caso

**Authors:** Ailton Carvalho Barbosa, Lívia Silva de Paula Faria, Larissa Freitas Peixoto Gloria, Graziella Viana da Silva, Paulo Henrique Ribeiro de Oliveira, Fabiano Vieira de Araujo, Felipe Eulalio Baldi Pessanha

**Affiliations:** 1 Hospital Estadual Ferreira Machado, Campos dos Goytacazes, RJ, Brasil; 2 Centro Universitário de Volta Redonda, Volta Redonda, RJ, Brasil

**Keywords:** COVID‐19, embolia pulmonar, trombose venosa profunda

## Abstract

Apesar de a fisiopatologia da coagulopatia associada à doença do coronavírus 2019 (COVID‐19) não ser bem conhecida, a ocorrência de embolia pulmonar (EP) é frequentemente observada. No entanto, foram descritos na literatura poucos casos de pacientes que tiveram COVID-19 oligossintomática, sem nenhum fator de risco para tromboembolismo venoso (TEV) e que apresentaram EP aguda extensa. Relatamos um caso de paciente com COVID-19 oligossintomática, complicada por trombose venosa profunda e, posteriormente, EP aguda extensa, sugerindo que esses quadros devem ser considerados de forma sistemática mesmo em pacientes com COVID-19 oligossintomática e sem fatores de risco conhecidos para TEV.

## INTRODUÇÃO

A pandemia da doença do coronavírus 2019 (COVID-19), causada pelo coronavírus 2 da síndrome respiratória aguda grave (SARS-CoV-2), tem provocado grande dificuldade ao sistema de saúde frente à ausência de conhecimento completo da doença, desde a sua etiopatogenia e complicações até o seu tratamento^1^. Apesar de a fisiopatologia da coagulopatia associada à COVID‐19 ainda não ser totalmente conhecida, já é sabido que o risco de a doença complicar com embolia pulmonar (EP) é alta, principalmente em casos graves ou críticos. O mecanismo da patogênese parece estar relacionado a um estado de hipercoagulação causado pela grande liberação de citocinas inflamatórias no organismo^2^. No entanto, a incidência de casos de EP aguda extensa como complicação da COVID-19 oligossintomática e sem fatores de risco conhecidos para tromboembolismo venoso (TEV) ainda não foi bem elucidada.

Neste relato de caso, descrevemos um paciente com suspeita de ter tido COVID-19 oligossintomática, sem fatores de risco conhecidos para TEV, que evoluiu com quadro de trombose venosa profunda (TVP) e, posteriormente, EP aguda extensa. Isso sugeriu a possibilidade de que esses quadros devam ser pensados de forma sistemática, mesmo em pacientes oligossintomáticos e sem fatores de risco conhecidos para TEV. Este estudo foi devidamente avaliado e aprovado pelo Comitê de Ética em Pesquisa (CAAE 43681721.3.0000.5237, protocolo 4.641.084).

## DESCRIÇÃO DO CASO

Um paciente do sexo masculino, com 57 anos de idade, sem história de hipertensão arterial sistêmica, diabetes melito ou outras comorbidades, procurou atendimento no pronto-socorro com queixa de dispneia leve e edema do membro inferior direito persistente. Relatou que o edema iniciou há aproximadamente 1 mês, tendo feito uso de compressas de gelo no local, e foi acompanhado de febre com calafrios por 3 dias consecutivos. O paciente estava afebril no momento do exame e negava história de tosse, cefaleia ou anosmia. Negava tabagismo, etilismo ou quaisquer cirurgias recentes.

Ao exame físico, o paciente apresentava bom estado geral, afebril, acianótico, dispneico em ar ambiente, e o seu índice de massa corpórea era de 27,78 kg/m^2^. A pressão arterial era 120×70 mmHg, a frequência cardíaca, de 90 bpm, a frequência respiratória, de 20 irpm e saturação de oxigênio, de 98% em ar ambiente. As auscultas respiratória e cardíaca estavam normais. O membro inferior direito apresentava edema (++/4+), sem empastamento muscular, e os pulsos eram palpáveis bilateralmente. Os exames laboratoriais solicitados apresentaram leucócitos 12.150 sem desvio para esquerda, hematócrito de 41%, CK-MB normal e eletrólitos, ureia e creatinina dentro dos padrões de normalidade.

O ecocardiograma, o eletrocardiograma e a radiografia de tórax não apresentaram alterações. Foi realizado eco-Doppler colorido de membros inferiores para elucidar a etiologia do edema, que evidenciou veia poplítea e solear direitas com sinais de trombose venosa aguda (veias incompressíveis, com material ecogênico intraluminal sem fluxo ao Doppler). Os exames D-dímero (DD) e proteína C-reativa (PCR) não estavam disponíveis naquele momento. Diante disso, para elucidar a etiologia da dispneia, foi realizada angiotomografia computadorizada de tórax (ATC), que não evidenciou sinais de pneumonia, mas identificou presença de trombo em artéria pulmonar bilateralmente (Figura 1) com áreas de infarto pulmonar à direita (Figura 2). Foi realizado também o teste rápido de COVID-19, sendo positivo para imunoglobina G (IgG) e negativo para imunoglobina M (IgM). Com base nesses achados, a hipótese diagnóstica foi de EP aguda extensa como complicação de COVID-19 oligossintomática.

**Figura 1:** Angiotomografia computadorizada de tórax, plano axial, evidenciando falha de enchimento completa em artérias pulmonares direita **(A)** e esquerda **(B)** devido à obstrução por trombo (setas).

**Figura 2:** Angiotomografia computadorizada de tórax, com janela para parênquima pulmonar, evidenciando área de infarto pulmonar à direita (seta).

Após avaliar o risco e o benefício em função da idade e doenças preexistentes, foi iniciada terapia trombolítica com Alteplase endovenosa, na dose inicial de 10 mg diluídos em 10 mL de soro fisiológico a 0,9% em infusão contínua por 5 minutos, seguidos de 90 mg diluídos em 90 mL de soro fisiológico a 0,9% venoso por 2 horas. O paciente permaneceu estável todo o tempo e sem instabilidade hemodinâmica, sem apresentar complicações devido ao uso do fibrinolítico. Ao final da administração de Alteplase, observou-se melhora da dispneia. Foi realizada uma nova ATC de tórax (Figura 3), 9 horas após a realização da primeira, a qual apresentou boa restauração do fluxo e resolução de grande parte do trombo.

**Figura 3:** Angiotomografia computadorizada de tórax, plano coronal, evidenciando restauração do fluxo sanguíneo em artérias pulmonares direita **(A)** e esquerda **(B)** e remoção de grande parte do trombo.

Ainda em vigência hospitalar, foi realizada uma monitorização hemodinâmica rigorosa do paciente, e, após 48 horas de internação, empregou-se dupla antiagregação com ácido acetilsalicílico 100 mg/dia e clopidogrel 75 mg/dia, não sendo iniciada antes devido ao risco de sangramento após o uso do fibrinolítico. O paciente recebeu alta após 6 dias de internação, pois, devido ao risco de reinfecção por SARS-CoV-2 e à necessidade de disponibilidade de leito para pacientes em estado grave, optou-se por não manter o paciente internado por período mais prolongado. Os ambulatórios oferecidos pelo Sistema Único de Saúde estavam suspensos por tempo indeterminado, e, por essa razão, não foi possível realizar o acompanhamento ambulatorial do paciente. No entanto, ele foi orientado a manter a dupla antiagregação e a retornar ao pronto-socorro caso apresentasse algum sinal de alarme ou piora clínica.

## DISCUSSÃO

O caso relatado destaca o risco de complicações tromboembolíticas em pacientes com diagnóstico de COVID-19, mesmo na sua forma oligossintomática, e a necessidade de ficar atento com esta possibilidade. No caso relatado, pelo fato de o paciente ter apresentado sinais e sintomas clássicos de TVP junto à dispneia, a investigação de EP era obrigatória. O escore de Wells pode ser usado para estimar o risco para pacientes com suspeita de TVP e EP e para ajudar no direcionamento referente aos exames a serem realizados em cada caso^3^. De acordo com a metanálise realizado por Suh et al.^4^, a taxa de incidência de EP e TVP em pacientes com COVID-19 foi de 16,5% e 14,8%, respectivamente. Entretanto, a TVP estava presente em apenas 42,4% dos pacientes com EP, sendo menor que a prevalência habitual em pacientes não infectados pelo SARS-CoV-2, que é de 60%^4^.

Em serviços públicos, hospitais como o do relato deste caso, a indisponibilidade de alguns exames, como o DD e o PCR, pode ser uma limitação para esclarecer a gravidade e o prognóstico do quadro de inflamação sistêmica causada pela COVID-19. Contudo, no caso relatado, a realização de ATC de tórax permitiu que o diagnóstico de EP fosse feito e que o tratamento fosse iniciado antes que o paciente evoluísse para uma instabilidade hemodinâmica ou óbito. Além disso, o teste rápido para a COVID-19 realizado indicou a infecção pelo SARS-CoV-2 como possível etiologia dessa complicação.

No que diz respeito ao tratamento, como a infecção pelo SARS-CoV-2 é uma doença nova, para a qual ainda não existe um tratamento comprovadamente eficaz, no caso em questão optou-se em tratar somente a EP a fim de evitar a progressão ou recidiva do quadro. Para auxiliar na decisão de qual o melhor tratamento a ser realizado, os pacientes devem ser avaliados quanto a gravidade do evento. O escore mencionado pela diretriz da Sociedade Europeia de Cardiologia de 2019 é o índice de gravidade da EP (PESI)^5^. A pontuação PESI do paciente relatado é de 67 pontos, o que o estratifica na categoria classe II, ou baixo risco, com uma mortalidade de 30 dias de 1,7 a 3,5%.

A pandemia da COVID-19 gerou um novo interesse em fibrinolíticos e seus possíveis benefícios no tratamento de pacientes com EP como complicação da infecção pelo SARS-CoV-2^2^. O uso desses agentes se justificaria pela reação de inflamação intensa e grande aumento de citocinas que ocorre na COVID-19, que leva à deposição de fibrina nos espaços aéreos e pulmonares, sendo um fator de gravidade no curso da doença^2^. Contudo, de acordo com a Sociedade Europeia de Cardiologia^5^, o uso desses agentes em pacientes de baixo risco é recomendado somente como terapia de resgate quando ocorre deterioração hemodinâmica apesar de ter sido feito tratamento prévio com anticoagulante.

No caso relatado, mesmo o paciente estando estável hemodinamicamente, a equipe médica optou por iniciar o tratamento com Alteplase pelos seguintes motivos: em primeiro lugar, a COVID-19 é uma doença nova e pode apresentar desfechos imprevisíveis e mórbidos, por isso preconizou-se pela resolução rápida da EP. O segundo motivo foi que o paciente apresentou na ATC uma EP extensa bilateralmente, para a qual o anticoagulante não seria tão eficiente quanto o fibrinolítico para a resolução dos trombos. Por fim, o paciente apresentava baixo nível socioeconômico e baixa escolaridade, não garantindo a continuidade do tratamento com anticoagulante em domicílio. Por esses motivos, após avaliação criteriosa dos riscos e benefícios, o uso do antifibrinolítico foi preconizado, sendo realizada monitorização rigorosa do paciente em vigência hospitalar.

Após um quadro de EP, os pacientes correm alto risco de recorrência caso não seja feita anticoagulação contínua. A diretriz do Colégio Americano de Médicos Torácicos^6^ recomenda o uso de anticoagulantes por pelo menos 3 meses após tratado o quadro de EP, com o período de uso variando de acordo com o risco de sangramento e/ou se os fatores que potencialmente contribuíram para a etiologia da EP ainda estão presentes. No entanto, para que isso seja realizado, é necessário um acompanhamento regular após a alta hospitalar para controle da razão normalizada internacional e para garantir que o paciente esteja aderindo ao tratamento ou que não tenha desenvolvido contraindicações à terapia. Conforme já relatado, como não seria possível o acompanhamento ambulatorial do paciente, optou-se por empregar somente a dupla antiagregação plaquetária com ácido acetilsalicílico e clopidogrel devido aos riscos que o uso do anticoagulante sem avaliação médica periódica poderia causar ao paciente.

Poucos casos de pacientes que tiveram COVID-19 oligossintomática, sem nenhum fator de risco conhecido para doença tromboembólica e que apresentaram EP aguda extensa foram descritos na literatura^7^^-^10. Portanto, salientamos que, enquanto a pandemia da COVID-19 estiver vigente, em casos já diagnosticados de infecção pelo SARS-CoV-2, sintomáticos ou assintomáticos, deve ser considerada a possibilidade de a doença complicar com alguma coagulopatia em casos admitidos no setor de emergência com TVP ou EP. Assim, principalmente em pacientes sem fatores de risco para doença tromboembólica, deve ser levantada a hipótese diagnóstica de ser uma complicação da COVID-19.
